# Oral Delivery of Avocado Peel Extract Using Albumin Nanocarriers to Modulate Cholesterol Absorption

**DOI:** 10.3390/pharmaceutics17081061

**Published:** 2025-08-15

**Authors:** Laura M. Teixeira, Ana S. Viana, Catarina P. Reis, Rita Pacheco

**Affiliations:** 1 Centro de Química Estrutural, Institute of Molecular Sciences, Faculdade de Ciências, Universidade de Lisboa, 1749-016 Lisboa, Portugal; fc57476@alunos.ciencias.ulisboa.pt (L.M.T.);; 2 Departamento de Química e Bioquímica, Faculdade de Ciências, Universidade de Lisboa, 1749-016 Lisboa, Portugal; 3 Institute for Medicines (iMed.ULisboa), Faculdade de Farmácia, Universidade de Lisboa, 1649-003 Lisboa, Portugal; 4 Instituto de Biofísica e Engenharia Biomédica (IBEB), Faculdade de Ciências, Universidade de Lisboa, 1749-016 Lisboa, Portugal; 5 Departamento de Engenharia Química, Instituto Superior de Engenharia de Lisboa, 1959-007 Lisboa, Portugal

**Keywords:** *Persea americana*, peel extract, drug delivery, nanoparticles, bioavailability, hypercholesterolemia

## Abstract

**Background/Objectives:** Hypercholesterolemia, a metabolic disorder and major risk factor for cardiovascular disease, remains a global health concern. Although current pharmacological interventions effectively reduce cholesterol levels, their use is often associated with adverse side effects. These limitations have driven interest in alternative or complementary approaches based on natural products; however, the poor solubility, stability, and bioavailability of many natural compounds emphasize the need for innovative drug delivery systems to enhance their health-promoting potential. The extract obtained from *Persea americana* peels, a sustainable and underutilized by-product, has previously been reported to have cholesterol-lowering properties. **Methods**: The extract was encapsulated in bovine serum albumin nanoparticles. The nanoformulation was characterized for physicochemical properties and for extract stability under acid-simulated gastric digestion. Safety and biocompatibility were evaluated by in vitro cytotoxicity assays using intestinal Caco-2 and liver HepG2 cells, and in vivo toxicity using *Artemia salina*. The bioavailability of the extract and the nanoformulation’s capacity to reduce cholesterol absorption in a differentiated Caco-2 cell model were additionally assessed. **Results**: Encapsulation enhanced extract stability and bioavailability, protecting it from degradation in acid simulated gastric digestion. The nanoparticles showed favorable physicochemical properties, including a small size of less than 100 nm, and demonstrated safety and biocompatibility. In the Caco-2 model, the encapsulation of the extract resulted in reduced cholesterol permeation compared to the free extract **Conclusions:** These findings suggest that the nanoformulation developed may offer a safe and effective strategy for the oral delivery of *P. americana* peel extract, reinforcing its potential for application in hypercholesterolemia management.

## 1. Introduction

Over the past few decades, nanotechnology has emerged as a powerful tool in the biomedical field, particularly in the development of advanced drug delivery systems (DDs). These nanocarriers offer several advantages over conventional formulations, including improved solubility, protection of compounds, controlled release, enhanced absorption, and targeted delivery to specific tissues. Such benefits are particularly relevant for the treatment of chronic diseases, where prolonged therapy and systemic side effects often reduce patient compliance and limit therapeutic efficacy [1,2,3].

Within this field, nanoparticles (NPs) have gained prominence as versatile DDSs [4,5]. Specifically, protein-based NPs have emerged as ideal candidates to use as a delivery system, as they are biodegradable, biocompatible, and generally non-toxic to the human organism [6,7]. Among them, NPs based on bovine serum albumin (BSA) have been widely studied due to their favorable safety profile and ease of preparation and purification, as well as their ability to encapsulate both hydrophilic and hydrophobic bioactive compounds [2,8,9]. BSA NPs offer multiple functional groups for chemical modification, facilitating surface functionalization with ligands for targeted delivery or with agents for theragnostic applications [10]. Furthermore, their ability to undergo controlled degradation under physiological conditions enables sustained and localized drug release, minimizing systemic toxicity [11]. Several studies have highlighted the potential of albumin-based nanocarriers in improving the pharmacokinetic profiles and therapeutic efficacy of anticancer agents, while also demonstrating favorable results in terms of cellular uptake and in vivo biocompatibility [12]. In addition to cancer therapy, BSA NPs have been investigated for applications in inflammatory diseases, neurological disorders, and antimicrobial delivery, illustrating their versatility as a nanoplatform [13]. The scalability and reproducibility of their synthesis, particularly using desolvation or emulsification techniques, further enhance their translational potential for clinical use [14]. These systems have shown great promise in enhancing the stability and bioavailability of therapeutic molecules, including synthetic drugs and natural compounds [15]. Moreover, the integration of natural products with advanced drug delivery systems is emerging as a promising strategy for addressing non-communicable diseases, such as cardiovascular diseases (CVDs), driven by the urgent need for more effective and safer therapeutic approaches [16].

To present, CVDs remain the leading cause of death globally, with hypercholesterolemia, a condition characterized by elevated blood cholesterol levels, being a major risk factor [17,18,19]. In this context, maintaining cholesterol levels within healthy ranges is essential to reduce the risk of CVDs. Some lifestyle interventions, such as a balanced diet low in saturated fats, are effective for some individuals [2,20]. Nevertheless, pharmacological interventions are necessary in more severe cases. Therefore, it is common to prescribe drugs such as statins, which inhibit 3-hydroxy-3-methylglutaryl coenzyme A reductase (HMGR), a key enzyme in hepatic cholesterol biosynthesis, and ezetimibe, which reduces intestinal cholesterol absorption by blocking the Niemann-Pick C1-Like 1 (NPC1L1) transporter [20,21,22,23]. Although effective, these pharmacological agents are frequently associated with adverse effects, including myopathy and hepatotoxicity, particularly with long-term use, thereby driving the search for safer and more sustainable alternatives [2,20]. In addition to their toxicity profile, statins exhibit poor oral bioavailability, often requiring an increase in daily doses to achieve therapeutic plasma levels. Likewise, ezetimibe shows limited solubility and low systemic uptake, further constraining its clinical efficacy [24,25].

More recently, nanotechnological strategies have also been applied to natural products to help overcome these limitations of prescribed drugs and additionally offer new perspectives for the development of safer and multifunctional complementary approaches [26]. Plant-derived food supplements, in particular, represent a rapidly expanding category, often perceived as safer and better tolerated than synthetic drugs [27,28,29]. These supplements normally incorporate bioactive compounds derived from natural origins such as peptides, vitamins, and phenolic compounds, including phenolic acids, flavonoids, and tannins, whose beneficial biological properties are well established [30,31]. The growing interest in bioactive compounds, particularly phenolic compounds, is attributed to their properties, such as antioxidant, antibacterial [32], anti-inflammatory [33], and cardioprotective effects [34], which support their role in preventing and managing various chronic diseases [35]. Notably, the antioxidant activity of phenolic compounds is especially important, as it helps protect cells from oxidative stress and damage caused by reactive species, which is recognized as a central mechanism underlying the onset and progression of various diseases and metabolic disorders, including diabetes and cardiovascular conditions [36,37].

Although these biological properties are often demonstrated, they do not always translate into the same effectiveness when these compounds are consumed. Upon consumption, natural bioactive compounds frequently face challenges such as poor solubility, chemical instability in the gastrointestinal tract, and reduced oral bioavailability, which significantly limit their therapeutic potential [2,38]. These limitations highlight the need for nanotechnological approaches that can improve the delivery and therapeutic efficacy of such compounds. Recent studies have demonstrated that encapsulating natural extracts into NPs, particularly polymeric-based ones, can significantly enhance their stability, enable controlled release, and improve absorption and bioavailability in the gastrointestinal environment [2,15].

One plant of growing interest in this context is *Persea americana*, commonly known as avocado, which has attracted growing interest due to its recognized health-promoting properties [39]. However, the processing of avocados generates a substantial volume of by-products, primarily peels and seeds, accounting for approximately 13–18% of the fruit’s dry mass [40,41,42]. Although often treated as agricultural waste, these by-products are abundant in valuable bioactive compounds such as phenolic compounds, which exhibit diverse biological activities with potential therapeutic applications [41,42,43]. Therefore, valorising these avocado residues presents an eco-friendly approach to obtain phenolic-rich extracts that can be incorporated into supplements and functional food products [43,44]. Our group has previously contributed to this valorisation by identifying the bioactive compounds present in an aqueous extract of *P. americana* peels and demonstrating their potential in managing hypercholesterolemia and promoting cardiovascular health [45,46].

Following these promising findings, the present study explores the incorporation of this extract into a polymeric-based drug delivery system using bovine serum albumin (BSA) NPs, aiming to enhance the stability and therapeutic efficacy of its major bioactive phenolic compounds, chlorogenic acid, catechin, and epicatechin. The resulting nanoformulations were characterized in terms of physicochemical properties, safety, and biological activity, with a particular focus on their potential anti-hypercholesterolemic effects, supporting their application as a novel, plant-based nanodelivery system for managing hypercholesterolemia.

## 2. Materials and Methods

### 2.1. Materials

#### 2.1.1. Chemicals

Dulbecco’s Modified Eagle Medium (DMEM), Roswell Park Memorial Institute Medium (RPMI-1640), and fetal bovine serum (FBS) were acquired from Biowest (Nuaillé, France). Cholesterol, sodium carbonate, bovine serum albumin (BSA), and 1,1-diphenyl-2-picrylhydrazyl (DPPH) were obtained from Sigma-Aldrich (Barcelona, Spain). Antimycotic and L-glutamine were purchased from Corning (New York, NY, USA). Glucose was acquired from HiMedia Laboratory (Mumbai, India). Calcium chloride, sodium bicarbonate, magnesium sulphate anhydrous, monopotassium phosphate, and hydrochloric acid (37% (*w*/*w*)) were purchased from Merck kGaA (Darmstadt, Germany). Disodium hydrogen phosphate and trifluoroacetic acid were obtained from PanReac (Barcelona, Spain). Dimethyl sulfoxide ultrapure (DMSO) and 3-(4,5-dimethylthiazol-2-yl)-2,5-diphenyl-tetrazolium bromide (MTT) were obtained from VWR (Radnor, PA, USA). Magnesium chloride hexahydrate, sodium chloride, and potassium chloride were acquired from Honeywell (Charlotte, NC, USA). Methanol was purchased from Fisher Scientific (Hampton, VA, USA). Ethanol absolute anhydrous was purchased from CARLO ERBA (Cornaredo, Italy). All chemicals were of analytical grade.

#### 2.1.2. Cell Culture

The human hepatocellular carcinoma (HepG2, ECACC 85011430) and human colorectal adenocarcinoma (Caco-2, ECACC 86010202) cell lines used in this study were acquired from the European Collection of Authenticated Cell Cultures. The Caco-2 cells were cultured in RPMI-1640 medium, and HepG2 cells in DMEM, both enriched with 10% fetal bovine serum (FBS), 2 mM L-glutamine, and antibiotics (penicillin 100 U/mL and streptomycin 100 U/mL). Cells were maintained in T75-flasks under standard culture conditions at 37 °C, 5% CO_2_, and the culture media were refreshed every 42–78 h; all assays were conducted between passages 3–10.

### 2.2. Methods

#### 2.2.1. Preparation of Unloaded and *Persea americana* Aqueous Extract-Loaded BSA NPs

The aqueous extract from peels from *P. americana* (Hass variety) was obtained following a previously reported methodology [46]. The peels of the avocado were washed and decocted in water (100 g/L) for 15 min. The resulting mixture was freeze-dried and the dried extract was stored at −20 °C until further use.

BSA NPs were produced following a modified version of the protocol reported by Santos-Rebelo et al. [15]. Briefly, 100 mg of BSA was dissolved in 4 mL of ultrapure water and the pH was adjusted from 7 to 10, with a 0.1 mM NaOH solution, where the final pH was 9. This solution was then introduced dropwise into 16 mL of absolute ethanol under continuous magnetic stirring at 500 rpm (Heidolph MR3001, Heidolph Instruments, Schwabach, Germany). Glucose (1.175 µL glucose/mg BSA) was then added to the suspension, which was kept under stirring for 30 min to ensure NP formation.

For extract-loaded NPs, 5, 10, and 15 mg of the dried extract were mixed with the BSA solution prior to pH adjustment, resulting in final extract concentrations of 0.25, 0.5, and 0.75 mg/mL, respectively, in the NPs suspensions. The final NPs suspensions were stored at 4 °C.

#### 2.2.2. Morphological Characterization of BSA NPs

Morphological characterization of unloaded and extract-loaded BSA NPs (5 mg) was performed using atomic force microscopy (AFM). Samples were diluted in a 1:4 ratio with a blank solution (20% ultrapure water–80% absolute ethanol), and 40 μL was added onto a mica surface. The samples were left to dry at room temperature for approximately 4 h. The analysis was conducted using a Multimode 8 HR instrument coupled to a Nanoscope V Controller (Bruker, Coventry, UK), operating with a scan rate of 1 Hertz, under peak force tapping mode with ScanAssist. Images were obtained using the ScanAsyst-air (0.4 N/m) (Bruker, Coventry, UK) tip model and processed with NanoScope V 1.8 software.

#### 2.2.3. In Vitro Gastric Digestion Simulation

For gastric digestion, the extract (0.05 and 0.55 mg/mL) and two extract-loaded BSA NPs samples (0.05 mg/mL) were used, one of which was in suspension and the other one was freeze-dried (Heto^®^, PowerDry LL3000, Milford, OH, USA). All of the samples were added to hydrochloric acid (HCl) at 0.01 M for two hours in an agitated, heated water bath (GLS Aqua 18 Plus, Grant, Cambridge, UK). Afterwards, the samples were collected and centrifuged (Z 233 M, HERMLE, Wehingen, Germany) for 20 min at 7500× *g*. The supernatant was collected and analyzed by means of HPLC-DAD using an Elite LaChrom^®^ (HITACHI, VWR, Tokyo, Japan), with an Autosampler L-2200, a Column Oven L-2300, and a Diode Array Detector L 2455 (VWR, Radnor, PA, USA). The column used was LiChroCART^®^ 250-4 LiCrosphere^®^100 RP-8 (5 μm) (Merk, Darmstadt, Germany), following the conditions described in a previous study [45]. The assays were carried out in duplicate, and for the extract, its AA was determined afterwards.

#### 2.2.4. In Vitro Safety Assays of the Extract-Loaded BSA NPs

For the in vitro assays, the NPs were first prepared in order to reduce the quantity of ethanol in the suspension (80%). Therefore, the NPs were centrifuged (Eppendorf 5415, Eppendorf^®^, Hamburg, Germany) for 20 min at 7500× *g*, and the supernatant was discarded. Afterwards, ultrapure water was added, and the process was repeated; the final concentration of ethanol was 1.8%.

Given this, the in vitro safety assays of the extract-loaded BSA NPs were performed using the 3-(4,5-dimethylthiazol-2-yl)-2,5-diphenyl-tetrazolium bromide (MTT) method, described by Mosmann [47]. Both cell lines, Caco-2 and HepG2, were incubated for 24 h with 100 µL solutions of the extract-loaded BSA NPs at different concentrations of extract (0.02–0.5 mg/mL). The cell viability was determined following Equation (1), where Abs_Sample_ corresponds to the absorbance of the sample and Abs_Control_ corresponds to the absorbance of the control. Additionally, 1% (m/V) SDS was used as a positive control, showing 0% viability. The assay was performed in quadruplicate.

(1) $$\mathrm{Cell}\;\mathrm{Viability}\;\left(\%\right)=\frac{{Abs}_{Sample}}{{\mathrm{Abs}}_{Control}}\;\times 100$$

#### 2.2.5. In Vivo Safety Assay of the Extract-Loaded BSA NPs

For the in vivo safety assay of the extract-loaded BSA NPs, the nanoformulations were also prepared in order to reduce the quantity of ethanol. Therefore, safety was assessed with *Artemia salina*, following the protocol described by Lopes et al. [48]. After hatching the dehydrated *A. salina* eggs (JBL Artemio Pur^®^ GmBh & Co., Neuhofen, Germany), the nauplii were collected and incubated with extracted-loaded BSA NPs (0.240 mg/mL) for 24 h. Artificial seawater was used as the negative control, while DMSO served as the positive control. Mortality was determined using Equation (2), where Dead*_A. salina_* corresponds to the number of dead *A. salina* after 24 h of being incubated with the sample and Total*_A. salina_* corresponds to the total *A. salina* added to each well. The assay was performed in quadruplicate.

(2) $$\mathrm{Mortality}\;\mathrm{Rate}\;\left(\%\right)=\frac{{\mathrm{Dead}}_{A.\;\mathrm{salina}}}{{\mathrm{Total}}_{A.\;\mathrm{salina}}}\;\times 100$$

#### 2.2.6. Antioxidant Activity of the Extract-Loaded BSA NPs

The antioxidant activity (AA) of the extract-loaded BSA NPs was determined following the 2,2-diphenyl-1-picrilhidrazil (DPPH) method, a procedure described by Coelho et al. [18]. The assays were performed in triplicate, and the (AA) was determined through Equation (3), where Abs_Sample_ corresponds to the absorbance of the sample and Abs_Control_ corresponds to the absorbance of the control. The assays were carried out in triplicate.

(3) $$\mathrm{Antioxidant}\;\mathrm{Activity}\;\left(\%\right)=\frac{{\mathrm{Abs}}_{\mathrm{Control}\;}-{Abs}_{Sample}}{{\mathrm{Abs}}_{\mathrm{Control}\;}}\;\times 100$$

#### 2.2.7. Cholesterol Permeability Analysis in a Gastrointestinal Model

For the cholesterol permeation study, the protocol described by Arantes et al. was followed [49]. For this purpose, in a 12-well microplate with inserts (A = 1.1 cm^2^), Caco-2 cells were cultured and maintained until differentiation. To verify the integrity of the membrane formed, its transepithelial electrical resistance (TEER) was evaluated, where the membrane was considered to be formed when the value was superior to 250 Ω·cm^2^.

Subsequently, on the basolateral side of each insert, 1.5 mL of Hank’s Balanced Salt Solution (HBSS) was added. On the apical side, 0.5 mL of each solution to be tested was added: cholesterol (5 mM) (control), extract-loaded BSA NPs (0.240 mg/mL) with cholesterol (5 mM), and ezetimibe (0.1 mM) with cholesterol (5 mM). The cells were maintained for 6 h at 37 °C in a 5% CO_2_ atmosphere. Afterwards, the basolateral solution was analyzed by means of HPLC-DAD with a LiChroCART^®^ 250-4 LiCrosphere^®^100 RP-8 (5 μm) column (Merk, Darmstadt, Germany), following the conditions presented by Pinto et al. [2].

Cholesterol reduction (%) was determined according to Equation (4), with the cholesterol amount detected after 6 h in the control group set as the 100% reference value, where Cholesterol_sample_ is the cholesterol on the basolateral side of the sample after 6 h of incubation, and Cholesterol_control_ is the cholesterol on the basolateral side of the control after 6 h. The assays were carried out in duplicate.

(4) $$\mathrm{Cholesterol}\;\mathrm{Reduction}\;(\%)=\frac{{\mathrm{Cholesterol}}_{\mathrm{Sample}}}{{\mathrm{Cholesterol}}_{\mathrm{Control}}}\;\times \;100$$

#### 2.2.8. Permeation Profile of Bioactive Compounds from the Extract

The permeation of the three phenolic compounds, previously identified in the extract, namely chlorogenic acid, catechin, and epicatechin [45], was evaluated for the extract-loaded BSA NPs. Therefore, for the preparation of the permeation assay, the protocol described in Section 2.2.7 was followed, where the sample studied was the extract-loaded BSA NPs (0.240 mg/mL) with cholesterol (5 mM). After 6 h of incubation, a sample of the basolateral side was collected and analyzed by means of HPLC-DAD with the same conditions described in Section 2.2.7.

The apparent permeability coefficient (P_app_) was determined using Equation (5), where V is the volume of the apical side in mL, C_i_ is the initial concentration on the apical side in mg/mL, A is the area of the insert membrane in cm^2^ (1.1 cm^2^), C_f_ is the concentration on the basolateral side in mg/mL and t is the time in seconds.

(5) $$P_{app}=\frac{V}{A\times C_{i}}\times \frac{C_{f}}{t}$$

#### 2.2.9. Statistical Analysis

The data is expressed as the mean ± standard deviation. Statistical differences were evaluated using analysis of variance, with a *p* = 0.05, (ANOVA) (Microsoft Office 365, Washington, DC, USA).

## 3. Results

The aqueous peel extract of *P. americana* was characterized by means of high-resolution mass spectrometry and high-performance liquid chromatography, confirming the presence of a diverse array of bioactive compounds. Among them, phenolic compounds such as chlorogenic acid, catechin, and epicatechin were identified as the major constituents, alongside other compounds such as caffeic acid and procyanidin B_2_. Additional bioactive compounds, including amino acids, fatty acids, and sugars, were also present. Quantitative analysis revealed a total phenolic content of 159.07 ± 0.02 mg GAE/g dry extract and strong in vitro antioxidant activity (EC_50_ = 6.0 ± 0.2 µg/mL in the DPPH assay) [45,46]. A brief summary of this characterization is provided in the Supplementary Materials Table S1.

Building on this compositional profile and demonstrated bioactive activities, the present work investigates the encapsulation of the extract’s bioactive compounds in BSA NPs, aiming to enhance their stability and improve bioavailability. In addition, the study evaluates whether these nanoformulations can modulate cholesterol permeation across a differentiated Caco-2 monolayer, used as an in vitro model of the intestinal barrier, considering that elevated intestinal cholesterol absorption contributes to increased blood cholesterol levels, a key factor in the development of hypercholesterolemia and related metabolic disorders such as atherosclerosis, metabolic syndrome, obesity, type 2 diabetes, and non-alcoholic fatty liver disease.

### 3.1. Characterizations of Extract-Loaded BSA NPs and Morphological Analysis

Different batches of BSA NPs were formulated, including unloaded NPs and NPs loaded with 5, 10, and 15 mg of extract. All NPs demonstrated an average hydrodynamic diameter below 400 nm, a polydispersity index (PdI) under 0.2, and a negative zeta potential. Additionally, the encapsulation efficiency determined for all nanoformulations was seen to be over 77%, however, achieving higher encapsulation efficiency for the BSA NPs loaded with 5 mg of extract (84%), which was chosen for all subsequent experiments [45,46]. A brief summary of this characterization is provided in the Supplementary Materials Table S2.

**Figure 1:**
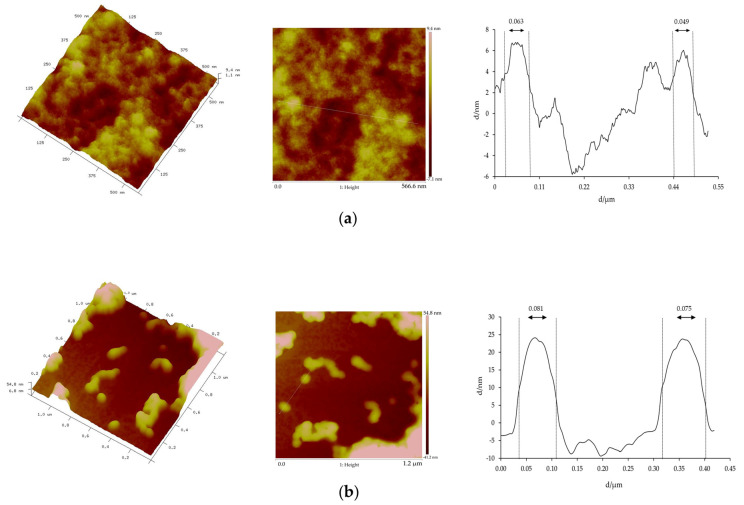
Three-dimensional AFM images and their corresponding height profiles of (**a**) unloaded and (**b**) extract-loaded BSA NPs.

The morphology of the prepared nanoformulations was characterized by means of Atomic Force Microscopy (AFM). The AFM images, presented in Figure 1, showed that both unloaded and extract-loaded BSA NPs displayed predominantly spherical morphology with individual particle sizes below 100 nm. A slightly larger mean particle size was observed for the extract-loaded BSA NPs compared to the unloaded ones. Some degree of particle aggregation was also evident, more pronounced in the unloaded BSA NPs. This tendency to aggregate may be likely due to the absence of extract compounds, which in the loaded NPs can reduce particle–particle interactions, leading to aggregation during the drying process for AFM sample preparation.

### 3.2. Gastric Digestion Simulation Study of the Extract and Extract-Loaded BSA NPs

A simulated gastric digestion assay was carried out to assess the stability of the extract and the formulated extract-loaded BSA NPs under simulated stomach conditions.

Initially, the extract was subjected to a 2 h gastric digestion process with a HCl solution (0.01 M). After digestion, HPLC-DAD was used to quantify the percentage of reduction in the amounts of the three major phenolic compounds in the extract: chlorogenic acid, catechin, and epicatechin. Additionally, the reduction in antioxidant activity (AA) of the extract after digestion was evaluated.

**Figure 2:**
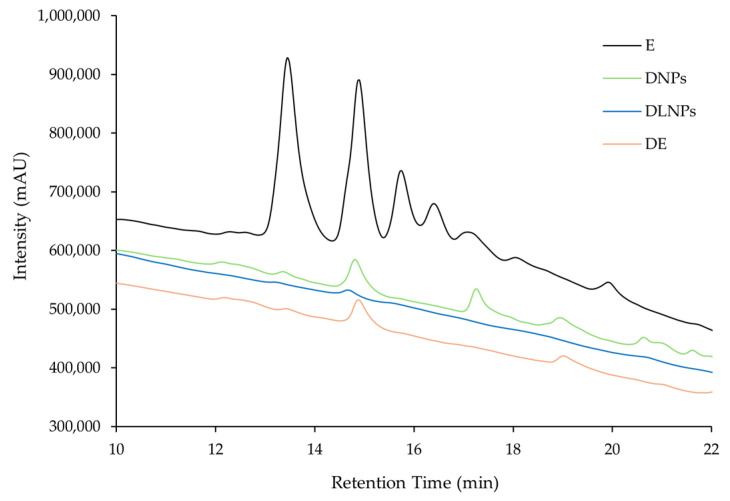
Chromatogram of the free extract (E), digested extract (DE), digested lyophilized NPs (DLNPs), and digested non-lyophilized NPs (DNPs), obtained by means of HPLC-DAD (10–22 min).

The results, presented in Table 1, revealed that gastric digestion led to a decrease of more than 55% in the content of each of the three phenolic compounds in the extract, which was associated with a 25% reduction in its antioxidant activity. As shown in Figure 2, the HPLC-DAD chromatograms of the extract before (E—black line) and after (DE orange line) simulated gastric digestion reveal an evident decrease in the levels of the compounds following digestion.

The extract-loaded BSA NPs were subjected to simulated gastric digestion. For this purpose, the nanoformulations were tested in both a suspension form and as a dry powder obtained through lyophilization of the extract-loaded BSA NPs to mimic potential consumption formats. After 2 h digestion in 0.01 M HCl, the samples were centrifuged, and the supernatant was analyzed by means of HPLC-DAD to assess the release of the extract compounds from the NPs.

As shown in Figure 2, the chromatogram of the digested non-lyophilized NPs (DNPs, green line) displayed distinct peaks corresponding to released extract compounds, whereas the digested lyophilized NPs (DLNPs, blue line) showed no detectable peaks. These results suggest that while both NP formulations can protect the encapsulated extract from acidic degradation, the non-lyophilized NPs release a portion of the bioactive compounds into the supernatant under simulated gastric conditions. In contrast, the lyophilized NPs appear to retain the extract more effectively, limiting its release during digestion. Overall, these findings indicate that BSA NPs can provide protection to the extract’s bioactive compounds during gastric transit.

### 3.3. In Vitro Safety Assay of Extract-Loaded BSA NPs

To assess the safety profile of the extract-loaded BSA NPs, cytotoxicity studies were performed on human-relevant in vitro models. Caco-2 and HepG2 human cell lines were selected to reflect the targeted oral route of administration. Caco-2 cells, similar to intestinal enterocytes, simulate the gastrointestinal tract, while HepG2 cells enable the assessment of potential hepatotoxicity. The use of these cell lines was employed as an approach to simulate the intestinal–hepatic axis to predict systemic safety.

The cells were exposed for 24 h to different concentrations of extract encapsulated into the BSA NPs.

**Figure 3:**
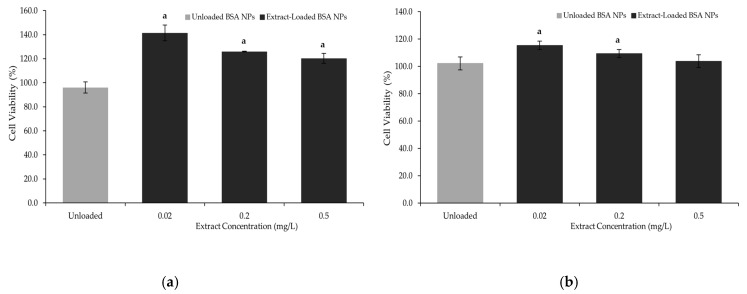
Cell viability (%) in (**a**) Caco-2 and (**b**) HepG2, after 24 h exposure to unloaded and extract-loaded BSA NPs at various concentrations, where “a” corresponds to samples that are statistically different from the unloaded BSA NPs, according to a one-way ANOVA (*p* ≤ 0.05).

From the results presented in Figure 3, it can be concluded that the cell viability remained above 90%, confirming the safety of the extract-loaded BSA NPs. These results demonstrate that the prepared nanoformulations showed no toxicity towards the tested human cell lines, which was also observed for the free extract in previous studies [45], suggesting that these NPs could be considered safe for human consumption.

### 3.4. In Vivo Safety Assay of Extract-Loaded BSA NPs

To complement the previous safety profile of the extract-loaded BSA NPs, an in vivo preliminary safety assay was carried out. *Artemia salina* was used as a model as it is used to evaluate the toxicity of chemicals, extracts, or drugs, as its responses can reflect potential toxic effects at the ecological level [50].

**Figure 4:**
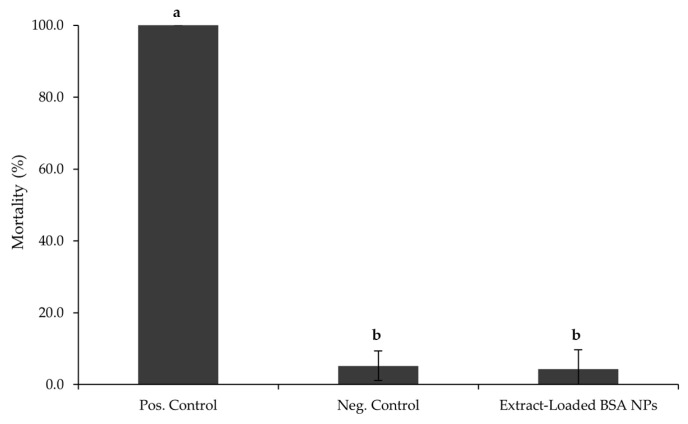
*A. salina* mortality (%) after 24 h of being incubated with the extract-loaded BSA NPs (0.240 mg/mL). The positive and negative controls were 100% DMSO and *Artemia* salt medium, respectively. Where “a” corresponds to samples that are statistically different from the negative control and “b” corresponds to samples that are statistically different from the positive control, according to a one-way ANOVA (*p* ≤ 0.05).

As presented in Figure 4, the mortality rate obtained for the NPs was lower than 5%, following 24 h of exposure to the nanoformulations, consistent with in vitro assays. These findings indicate that the formulated NPs do not have any impact on the environment. Overall, the safety data from both the MTT cytotoxicity assay and *Artemia salina* lethality test support the biocompatibility of the extract-loaded BSA NPs, reinforcing their potential as a safe candidate for use in food supplements or nutraceutical applications and environmentally friendly when compared to other pharmaceuticals.

### 3.5. Antioxidant Activity of Extract-Loaded BSA NPs

**Figure 5:**
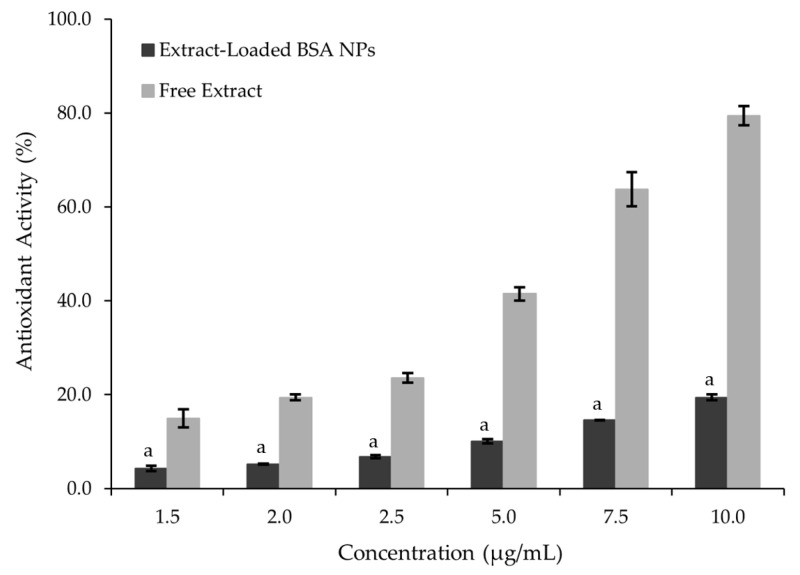
Antioxidant activity (AA) (%) of the free extract and extract-loaded BSA NPs for different concentrations. At each concentration, bars labeled with “a” are statistically different from the free extract, according to a one-way ANOVA (*p* ≤ 0.05).

The antioxidant activity (AA) of the extract-loaded BSA NPs was evaluated using the DPPH radical scavenging assay across different concentrations and compared to the same concentrations of the free extract. As shown in Figure 5, the encapsulated extract exhibited a notably lower AA than the free extract at equivalent concentrations. This reduction in measured AA after encapsulation has also been reported in previous studies on polymeric NPs containing natural extracts [2,8]. One key explanation is the limited availability of active phytochemicals, such as polyphenols and flavonoids, at the surface of the NPs, which restricts their immediate interaction with free radicals in standard in vitro assays. Furthermore, encapsulation often alters the molecular conformation or reduces the extract’s direct exposure to the aqueous environment, thereby hindering radical-scavenging reactions. In some cases, the protein structure of BSA can also act as a diffusion barrier, delaying the release of active compounds, especially in non-physiological conditions used in in vitro antioxidant testing.

### 3.6. Effect of Extract-Loaded BSA NPs on Intestinal Cholesterol Permeation

The potential of the extract-loaded BSA NPs to reduce intestinal cholesterol absorption was investigated using a well-established in vitro model of differentiated Caco-2 cell monolayers. Caco-2 cells are widely recognized for their ability to mimic the morphological and functional characteristics of enterocytes in the intestinal lining, making them a reliable tool for simulating intestinal bioavailability [51].

Therefore, the cells were exposed to the extract-loaded BSA NPs at a concentration of 0.240 mg/mL. To mimic the physiological conditions of exposure to dietary cholesterol, a 5 mM cholesterol solution was applied to the apical side of the insert. Cholesterol permeation was assessed by quantifying the amount that crossed the monolayer in the presence of the NPs with that of a control group treated only with the cholesterol solution (100% permeability). Additionally, an assay in the presence of ezetimibe, a clinically prescribed drug that inhibits cholesterol absorption in the intestinal barrier, was used as a positive control.

**Figure 6:**
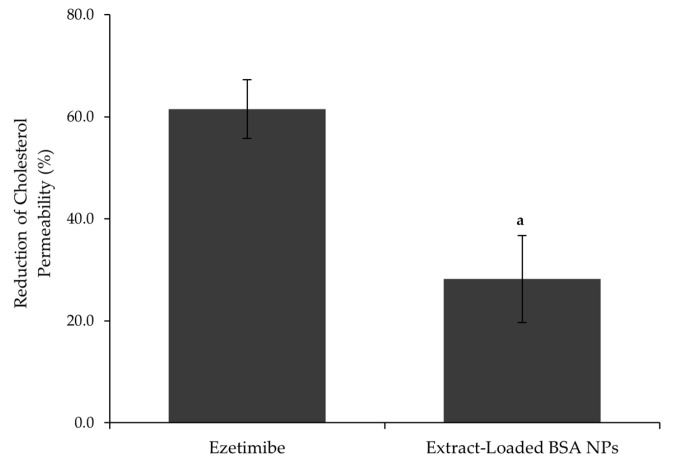
Reduction in cholesterol permeability (%) for ezetimibe (0.01 mM) and extract-loaded BSA NPs (0.240 mg/mL), where “a” corresponds to the sample that is statistically different from the ezetimibe, according to a one-way ANOVA (*p* ≤ 0.05).

As shown in Figure 6, extract-loaded BSA NPs reduced cholesterol permeation by 28 ± 9%. While ezetimibe achieves a higher reduction of 62 ± 5% under the same conditions, the extract-loaded BSA NPs demonstrated a significant inhibitory effect, especially considering it is a natural derived complex mixture of bioactive compounds.

Previous studies on the free extract alone showed a cholesterol permeability reduction of 22 ± 6% [45,46]. Thus, encapsulation in BSA NPs enhanced the extract’s efficacy, representing approximately a 1.3-fold improvement over the free extract.

These results highlight the promising potential of the extract-loaded BSA NPs as a natural complementary approach for cholesterol management.

The intestinal bioavailability of the extract’s major phenolic compounds, chlorogenic acid, catechin, and epicatechin was additionally evaluated in the permeability assay and the apparent permeability coefficients (P_app_) of these compounds when encapsulated in the extract-loaded BSA NPs were determined.

As shown in Table 2, only catechin and epicatechin were able to permeate the Caco-2 monolayer, with Papp values of 2.9 × 10^−6^ cm/s and 4.2 × 10^−6^ cm/s, respectively. According to established criteria, compounds with P_app_ values below 1.0 × 10^−6^ cm/s are classified as having low permeability, those with values between 1.0 × 10^−6^ cm/s and 10.0 × 10^−6^ cm/s correspond to moderate permeability, and those with values above 10.0 × 10^−6^ cm/s indicate high permeability [52]. Thus, both catechin and epicatechin demonstrated moderate permeability under these conditions.

Compared to previously reported results using the free extract under the same conditions [45,46], catechin permeability improved significantly, as it was not detected in the free extract permeability assay. Conversely, epicatechin permeability decreased slightly from high to moderate when encapsulated, suggesting a possible variation of its transport dynamics when loaded to the NPs.

## 4. Discussion

In recent years, nanotechnology-based DDS has emerged as a promising strategy to improve the therapeutic applications of natural products [26]. Given the growing interest in natural products as safer alternatives or complements to conventional therapies, particularly for conditions like hypercholesterolemia and CVDs, such delivery systems can significantly improve their efficacy and bioavailability [1,2,3] while minimizing adverse side effects associated with current pharmacological treatments [20].

In this context, the present work reports the successful development of BSA NPs encapsulating an aqueous extract of avocado peels, rich in bioactive compounds. The nanoformulations demonstrated improved physicochemical stability, protection of bioactive compounds from degradation, controlled release, and enhanced intestinal permeability of catechin. Moreover, the extract-loaded BSA NPs reduced cholesterol permeation across the intestinal lining model compared to the free extract. These results highlight the potential of this plant-based nanodelivery system as a complementary strategy for managing hypercholesterolemia and related metabolic disorders.

The extract-loaded BSA NPs developed were characterized by means of DLS [45] and AFM. DLS provides the hydrodynamic diameter of particles in suspension, including solvation layers and dynamic aggregates, thus reflecting behaviour closer to in vivo conditions. In contrast, AFM analyzes dried particles on a substrate, typically yielding smaller sizes due to the absence of hydration and occurrence of particle–particle interactions and aggregation during drying. Notably, AFM images showed some degree of particle aggregation, more pronounced in the unloaded BSA NPs, likely due to the absence of extract compounds. In contrast, the extract-loaded NPs exhibited reduced aggregation after the AFM drying process, possibly as a result of the extract compounds reducing particle–particle interactions. However, this aggregation observed in AFM did not reflect the true state of the NPs in suspension. DLS measurements confirmed that both types of NPs maintained good colloidal stability in aqueous media, with low PdI and consistent size distribution. The reduced aggregation in extract-loaded NPs suggests improved stability and potential enhanced performance, as particle stability is essential to maintain dispersion in the gastrointestinal tract and promote absorption. Together, these complementary techniques confirmed the nanoscale dimensions and morphology of the developed NPs.

Particle size plays a crucial role in oral delivery and absorption. Particles under 500 nm are generally considered favourable for oral delivery, as they can be taken up by M cells in Peyer’s patches or via transcytosis in intestinal epithelial cells. Also, NPs with sizes below 300 nm can prevent enterocytes from absorbing substances [53], while those under 100 nm may benefit from prolonged circulation and reduced hepatic clearance [54]. These findings suggest that the extract-loaded BSA NPs developed exhibit promising physicochemical characteristics for effective oral delivery and systemic circulation. The relatively small hydrodynamic sizes, below 400 nm, and negative zeta potential of these NPs [45], suggest good colloidal stability in aqueous media, which is essential for maintaining dispersion in the gastrointestinal tract and promoting absorption. Furthermore, the nanoscale dimensions observed by means of AFM support potential mucosal adhesion and interaction with enterocytes, enhancing intestinal uptake. Additionally, the PdI values obtained for these NPs were below 0.2, indicating a narrow size distribution, reflecting a homogeneous population of particles. Furthermore, the encapsulation efficiency of the extract-loaded BSA NPs was consistently above 77%, reaching up to 84% for the formulation containing 5 mg of extract. This high encapsulation demonstrates the strong affinity and effective incorporation of the bioactive compounds within the BSA matrix, aligning with previous reports on BSA-based delivery systems [2]. High encapsulation efficiency is critical to ensure sufficient payload delivery to the target site and can contribute to enhanced therapeutic outcomes. These high encapsulation efficiency values suggest a strong interaction between the bioactive compounds and the BSA matrix, likely due to hydrophobic interactions and hydrogen bonding, as commonly observed in albumin-based systems [55]. Such efficient encapsulation is advantageous not only for maximizing drug loading but also for ensuring sustained release and therapeutic efficacy. Moreover, our findings are in agreement with previously reported studies on BSA NPs, which have demonstrated similar encapsulation potential for various hydrophobic compounds [56].

The in vitro simulated gastric digestion assays further demonstrated the protective role of BSA NPs as nanocarriers of the extract. While the free extract was notably degraded under gastric conditions, encapsulation within the BSA matrix conferred protection against degradation and enabled a controlled release of its bioactive compounds, showing strong potential as a delivery system. This behaviour suggests a pH-responsive or enzyme-triggered release mechanism, consistent with the known stability and digestibility profile of albumin-based carriers [57]. Notably, the absence of peaks in the chromatogram of the supernatant from the lyophilized digested extract loaded BSA NPs indicates stronger protection, but limited release, possibly due to structural changes induced by NP lyophilization. This latter effect should be further investigated in future studies, particularly if the intended mode of consumption or administration involves the use of lyophilized formulations.

The MTT cytotoxicity assay and the *Artemia salina* lethality test support the biocompatibility of the extract-loaded BSA NPs. Combined with the use of natural, upcycled plant extracts and biodegradable carriers, this strategy reinforces their potential as a safe and environmentally sustainable alternative to synthetic drugs for food supplements or nutraceutical applications. Additionally, the enhanced cell viability observed upon exposure to the extract-loaded BSA NPs may be attributed to the growth-promoting or other biological activities of the extract’s phytochemicals. These findings not only confirm the non-toxic nature of the formulation but also point to a potential therapeutic advantage of encapsulating such bioactives within a protein-based delivery system.

The reduction in measurable AA on the extract-loaded BSA NPs relative to the free extract was likely due to the retention of bioactive compounds within the NP matrix, limiting their immediate interaction with assay reagents. Nevertheless, such an outcome supports the successful encapsulation of the bioactives, as their reduced availability in solution implies strong association with the BSA carrier.

Importantly, the extract-loaded BSA NPs significantly reduced cholesterol permeability across the intestinal model relative to the free extract, suggesting improved bioactivity and bioavailability, likely driven by increased stability and cellular uptake.

In addition, while encapsulation slightly reduced the apparent permeability of epicatechin from high to moderate, relative to the free extract, the nanoformulation offered clear benefits in protecting and improving catechin transport, highlighting the formulation’s potential to modulate the intestinal absorption of phenolics. The moderate permeability of both catechin and epicatechin also indicates their potential to reach the systemic circulation and target tissues, such as the liver, where they may contribute to cholesterol regulation by inhibiting enzymes like HMG-CoA reductase.

Collectively, these findings emphasize the promise of the *P. americana* peel extract encapsulated within BSA NPs as a natural, multifunctional strategy to manage hypercholesterolemia.

Nevertheless, future research is necessary to fully realize their therapeutic potential as complementary or alternative interventions for cardiovascular health.

The precise mechanism underlying the demonstrated effects remains to be fully elucidated. Intestinal cholesterol absorption involves several key pathways, including the NPC1L1 transporter, scavenger receptor class B type I (SR-BI), and ATP-binding cassette transporters such as ABCG5/G8, among others [20]. Although some plant-derived bioactives, including polyphenols and phytosterols, have been reported to modulate these pathways, such as NPC1L1 and ABCG5/G8 expression, the present study did not include mechanistic assays to investigate these pathways [58].

Furthermore, in vivo studies are important to validate the hypocholesterolemic activity of the extract-loaded BSA NPs and assess their long-term efficacy and safety. Animal models, such as C57BL/6 mice or Wistar rats subjected to diet-induced hypercholesterolemia, could provide valuable insights as they mimic human metabolism.

In addition, the potential inherent effects of BSA on cholesterol and phenolic compound absorption are important to consider. BSA is known to bind a variety of molecules, potentially influencing their permeability and uptake in the intestine. Some studies suggest that albumin can modulate lipid transport and metabolism [59], possibly contributing synergistically to the cholesterol-lowering effects observed with the extract-loaded BSA NPs. Future research should also include comparisons with alternative nanocarriers (e.g., poly(lactic-co-glycolic acid), chitosan, and lipid-based systems) in terms of encapsulation efficiency, physicochemical stability, release kinetics, and biological performance.

## 5. Conclusions

Given the well-documented limitations and adverse effects associated with many conventional lipid-lowering therapies, NP-based DDSs have gained increasing attention as alternative platforms, particularly for enhancing the delivery and efficacy of natural compounds with therapeutic potential.

In this study, bioactive-rich extracts derived from *P. americana* (avocado) peels were successfully encapsulated into BSA NPs. The resulting nanoformulations exhibited favorable physicochemical characteristics, including optimal size, stability, and encapsulation efficiency, and demonstrated good biocompatibility in both in vitro and in vivo models. Importantly, the extract-loaded BSA NPs significantly improved the extract’s efficacy in reducing intestinal cholesterol permeability, highlighting the potential of BSA NPs as a complementary nutritional or therapeutic strategy for managing hypercholesterolemia and thereby mitigating the risk of CVDs.

Furthermore, the demonstrated ability of the NPs to shield the bioactive compounds from degradation under simulated digestive conditions underscores their utility as a promising oral delivery nanocarrier, especially within the context of food supplements.

To build on these findings, further research is ongoing to elucidate the underlying mechanisms by which the encapsulated extract modulates cholesterol absorption, such as identifying specific bioactive constituents responsible for this effect and characterizing their interactions with key intestinal transporters (e.g., NPC1L1) and metabolic enzymes (e.g., ACAT2 and CYP450 isoforms). Such insights will be critical for optimizing formulation strategies and validating the clinical relevance of this delivery system in lipid metabolism modulation.

In conclusion, this study provides an encouraging foundation for the development of bio-based nanocarriers for lipid-lowering interventions. Continued research will focus on understanding molecular mechanisms, optimizing targeted delivery, and validating efficacy through in vivo models to fully establish the potential of this nanoformulation in cardiovascular disease management.

## Figures and Tables

**Table 1:** Percentage of reduction in the quantity of chlorogenic acid, catechin, epicatechin, and antioxidant activity (AA) of the extract after a 2 h simulated gastric digestion process.

Compounds	Reduction in Quantity (%)
Chlorogenic Acid	69.5 ± 1.4
Catechin	63.5 ± 0.1
Epicatechin	56.6 ± 1.5
AA	25.1 ± 0.7

**Table 2:** Apparent permeability coefficient (P_app_) for the three phenolic compounds identified in the extract-loaded BSA NPs. N.D., not detected.

Sample	Compound	P_app_ (10^−6^ cm/s)
Extract-Loaded BSA NPs + Cholesterol	Chlorogenic Acid	N.D.
	Catechin	2.9
	Epicatechin	4.2

## Data Availability

The original contributions presented in this study are included in the article/Supplementary Materials. Further inquiries can be directed to the corresponding authors.

## Supplementary Materials

The following supporting information can be downloaded at https://www.mdpi.com/article/10.3390/pharmaceutics17081061/s1. Table S1. Characterization of the *Persea americana* peel extract. Adapted from [46]; Table S2. Characterization of the extract-loaded BSA NPs. Adapted from [45].

## Abbreviations

The following abbreviations are used in this manuscript:

AFM	Atomic Force Microscopy
BSA	Bovine Serum Albumin
CVD	Cardiovascular Disease
DDS	Drug Delivery System
DE	Digested Extract
DLNPs	Digested Lyophilized Nanoparticles
DNPs	Digested Non-Lyophilized Nanoparticles
DLS	Dynamic Light Scattering
E	Extract
GAE	Gallic Acid Equivalents
HMGR	3-hydroxy-3-methylglutaryl coenzyme A reductase
HPLC-DAD	High-Performance Liquid Chromatography Coupled to a Diode-Array Detector
NPC1L1	Niemann-Pick C1-Like 1
NPs	Nanoparticles
TPC	Total Phenolic Content
P_app_	Apparent Permeability Coefficient
