# Supplementary material for: Oral Delivery of Avocado Peel Extract Using Albumin Nanocarriers to Modulate Cholesterol Absorption
Source: Pharmaceutics. 2025 Aug 15;17(8):1061. doi: 10.3390/pharmaceutics17081061 (PMC12389412; doi:10.3390/pharmaceutics17081061)
Supplement: Supplementary file 1 [file pharmaceutics-17-01061-s001.zip › pharmaceutics-3799474-SI.pdf]

Article

Oral Delivery of Avocado Peel Extract Using Albumin Nanocarriers to Modulate Cholesterol Absorption

Laura M. Teixeira [1,2,3], Ana S. Viana [1,2], Catarina P. Reis [3,4,*] and Rita Pacheco [1,5,*]

[1] Centro de Química Estrutural, Institute of Molecular Sciences, Faculdade de Ciências,
Universidade de Lisboa, 1749-016 Lisboa, Portugal;
fc57476@alunos.ciencias.ulisboa.pt (L.M.T.);
apsemedo@ciencias.ulisboa.pt (A.S.V.)

[2] Departamento de Química e Bioquímica, Faculdade de Ciências, Universidade de Lisboa,
1749-016 Lisboa, Portugal

[3] Institute for Medicines (iMed.ULisboa), Faculdade de Farmácia, Universidade de Lisboa,
1649-003 Lisboa, Portugal

[4] Instituto de Biofísica e Engenharia Biomédica (IBEB), Faculdade de Ciências, Universidade de Lisboa,
1749-016 Lisboa, Portugal

[5] Departamento de Engenharia Química, Instituto Superior de Engenharia de Lisboa,
1959-007 Lisboa, Portugal

* Correspondence: catarinareis@ff.ulisboa.pt (C.P.R.); rita.pacheco@isel.pt (R.P.)

Table S1. Characterization of the *Persea americana* peel extract. Adapted from [45].

Bioactive Compounds Identified	Chlorogenic acid, Catechin, Epicatechin, Caffeic acid, Procyanidin B2, mucic acid, Quinic acid, Citric acid, Choline cation, Pyridoxine + O-Hex, L-Glutamine, Perseitol, L-Tyrosine, Glutathione, Adenosine, Palmitamide, Oleamide, Octadecanamide.
Chlorogenic Acid (mg/g of dry extract)	18.60 ± 0.01
Catechin (mg/g of dry extract)	73.24 ± 0.01
Epicatechin (mg/g of dry extract)	83.14 ± 0.02
Total Phenolic Content (mg GAE/g dry extract)	159.07 ± 0.02
Antioxidant Activity (EC_{50} μg/mL)	6.0 ± 0.2

Table S2. Characterization of the extract-loaded BSA NPs. Adapted from [44].

BSA NPs	Mean Size (nm)	PdI	Zeta Potential (mV)	EE (%)
Unloaded	220 ± 10	0.151 ± 0.023*	−24 ± 4	-
5 mg of extract	275 ± 20	0.170 ± 0.009*	−25 ± 7	84 ± 5
10 mg of extract	351 ± 26	0.135 ± 0.044*	−30 ± 13	81 ± 1
15 mg of extract	401 ± 5	0.132 ± 0.001*	−36 ± 1	77 ± 4

*ISO 22412:2017 standard methods for determining particle size distributions using dynamic light scattering (DLS). According to this guideline, a PdI value below 0.2 is indicative of a monodisperse or narrowly distributed particle

population, ensuring consistency and stability of the formulation. Moreover, the ISO/TR 13014:2012 technical report emphasizes the importance of reporting particle size and distribution as critical quality attributes for nanomaterials intended for medical use, particularly when assessing their safety, stability, and efficacy.
